# Geographic Variation in Process and Outcomes of Care for Patients With Acute Myocardial Infarction in China From 2001 to 2015

**DOI:** 10.1001/jamanetworkopen.2020.21182

**Published:** 2020-10-23

**Authors:** Qi Zhong, Yan Gao, Xin Zheng, Jiyan Chen, Frederick A. Masoudi, Yuan Lu, Yingqing Feng, Shuang Hu, Qiuli Zhang, Cheng Huang, Yun Wang, Harlan M. Krumholz, Xi Li, Yingling Zhou

**Affiliations:** 1 Department of Cardiology, Guangdong Cardiovascular Institute, Guangdong Provincial Key Laboratory of Coronary Disease, Guangdong Provincial People’s Hospital, Guangdong Academy of Medical Sciences, Guangzhou, China; 2 National Clinical Research Center of Cardiovascular Diseases, State Key Laboratory of Cardiovascular Disease, Fuwai Hospital, Chinese Academy of Medical Sciences and Peking Union Medical College, National Center for Cardiovascular Diseases, Beijing, China; 3 NHC Key Laboratory of Clinical Research for Cardiovascular Medications, Beijing, China; 4 Division of Cardiology, University of Colorado Anschutz Medical Campus, Aurora, Colorado; 5 Center for Outcomes Research and Evaluation, Yale-New Haven Hospital, New Haven, Connecticut; 6 Section of Cardiovascular Medicine, Department of Internal Medicine, Yale School of Medicine, New Haven, Connecticut; 7 Department of Health Policy and Management, Yale School of Public Health, New Haven, Connecticut; 8 Central China Subcenter of the National Center for Cardiovascular Diseases, Zhengzhou, China; 9 Zhuhai Golden Bay Center Hospital, Guangdong Provincial People’s Hospital

## Abstract

**Importance:**

Variations across regions for managing acute myocardial infarction (AMI) in China are little understood.

**Objective:**

To evaluate geographic variation and its change with time in treatment process and outcomes for patients with AMI.

**Design, Setting, and Participants:**

This cross-sectional study used data from the Patient-Centered Evaluative Assessment of Cardiac Events–Retrospective AMI project in 2001, 2006, 2011, and 2015 in 153 randomly selected hospitals across China. Patients were hospitalized for AMI. Data were analyzed from October 1 to October 31, 2019.

**Exposures:**

Hospitalization in 3 geographic regions (Eastern, Central, and Western) stratified according to China’s official definition.

**Main Outcomes and Measures:**

Process of care measures included reperfusion therapies, aspirin, clopidogrel, β-blockers, angiotensin-converting enzyme inhibitors or angiotensin receptor blockers, and statins. Therapy use was analyzed among patients who were clinically eligible without contraindications (considered as ideal candidates for treatments). Outcome measures included in-hospital mortality and 5-day mortality. Mixed models were used to assess the regional disparities and time-region interactions in those measures, adjusting for patient characteristics.

**Results:**

In 153 hospitals across China, 27 046 patient hospitalizations for AMI were sampled. There was a significant difference across regions in process of care and the odds ratio (OR) of delivering any 1 of the 6 treatments to an ideal patient was 0.83 (95% CI, 0.76-0.91; *P* < .001) for the lowest region compared with the highest region. The variation between the 2 higher regions narrowed (time-by-Eastern region interaction: OR, 0.83; 95% CI, 0.76-0.91; *P* < .001). The region with the highest in-hospital mortality had 1.46 times greater in-hospital mortality (95% CI, 1.07-2.00; *P* < .001) than the lowest region and the region with the highest 5-day mortality had 1.52 times greater 5-day mortality (95% CI, 1.09-2.11; *P* = .04) than the lowest region. The geographic variation in mortality did not change over time.

**Conclusions and Relevance:**

In this study, significant geographic variations in process of care and outcomes were found to persist in China; further targeted and region-based approaches to AMI management are warranted.

## Key Points

**Question:**

What are the variations in process of care and outcomes for patients with acute myocardial infarction in China by geographic area?

**Findings:**

In this cross-sectional study of 27 046 hospitalizations from 2001 to 2015, patients in the region with worst performance were 17% less likely to receive optimal treatments comparing with the region with the best performance. Patients experienced a higher risk of in-hospital death by 46% in the region of highest mortality rate than in that of lowest mortality rate.

**Meaning:**

In this study, geographic variation in care delivery and in-hospital outcomes persisted, suggesting the need for more targeted research and investment in different geographic regions.

## Teaser

This cross-sectional study evaluates geographic variation and its change with time in treatment process and outcomes for patients with acute myocardial infarction in China.

## Introduction

The burden of cardiovascular disease in China has increased during the past 2 decades. Between 2002 and 2015, mortality of acute myocardial infarction (AMI) increased from 28.5 per 100 000 people to 126.5 per 100 000 people.^1^ Timely delivery of evidence-based in-hospital treatments^2,3,4,5,6,7,8^ has been considered as essential for positive outcomes after acute cardiac events.^9,10^ Therefore, close monitoring of the quality of care for patients with AMI has become a priority.

Providing high-quality care to all individuals in different regions is challenging, especially in a country such as China with varied geographic settings. Economic status and regional practices may impact the treatment patterns and outcomes.^11,12^ China developed national programs to support and facilitate the improvement of the quality of care. In 2009, a national health care reform was launched to promote access to and quality of health care across the country.^13^ Data from the 2003 to 2011 China National Health Services Surveys confirm increased health care use and coverage and advances in achieving equal access to health services across regions.^14^ However, little is known regarding geographic variation and its temporal trends in the quality of care in China, particularly under the favorable policy for underdeveloped region (ie, the Western) to mitigate the regional disparities.^15^ Understanding regional pattern of care in China may benefit the government’s layout planning and reform.

Accordingly, we assessed geographic variation in AMI care across regions of China before and after the launch of health care reform, using the China Patient-centered Evaluative Assessment of Cardiac Events Retrospective Study of Acute Myocardial Infarction (China PEACE-Retrospective AMI Study), a nationally representative sample of AMI hospitalizations.^16^ We estimated the variations in delivery of guideline-recommended treatments and in-hospital outcomes across regions, and evaluated how the variations change.

## Methods

### Study Design and Sample

Details of the China PEACE-Retrospective AMI Study design have been described previously.^16^ In brief, the study used stratified 2-stage random sampling to obtain representative samples of AMI hospitalizations in mainland China in 2001, 2006, 2011, and 2015. As hospital volumes and clinical capacities differ between urban and rural areas as well among the 3 official economic-geographic regions of Mainland China, we separately identified hospitals in 5 strata: Eastern-rural, Central-rural, Western-rural, Eastern-urban, and Central/Western-urban regions. In the first stage, we identified hospitals using a simple random sampling procedure within each of the 5 study strata. In the second stage, we drew cases based on the local hospital database for patients with AMI at each sampled hospital using systematic random sampling procedures. *International Classification of Diseases, Ninth Revision (ICD-9)* (410.xx) and *International Statistical Classification of Diseases and Related Health Problems, Tenth Revision (ICD-10)* (I21.xx) were used to identify patients with a principal discharge diagnosis of AMI. We excluded patients whose AMI occurred during the course of the hospitalization, those who transferred in or out, or were discharged alive within the first 24 hours of admission. We doubled the sample sizes for 2011 and 2015, to improve precision in the description of hospital-level treatment patterns and outcomes. Detailed clinical data were collected by central abstraction of medical records using standardized data definitions. Rigorous monitoring was conducted at each stage to ensure data quality, with an overall accuracy exceeded 98%. This study followed the Strengthening the Reporting of Observational Studies in Epidemiology (STROBE) reporting guideline for cross-sectional studies.

The central ethics committee at the National Center for Cardiovascular Diseases approved the study and waived the requirement for informed consent because of the retrospective nature of the study. All collaborating hospitals either accepted the central ethics approval or obtained local approval from an internal ethics committee.

### Patient and Hospital Characteristics

Trained staff abstracted the following patient characteristics: age, sex, cardiovascular risk factors (current smoker, hypertension, diabetes, and dyslipidemia), medical history (prior coronary heart disease, myocardial infarction, and stroke), and clinical profile at hospital presentation (systolic blood pressure, heart rate, white blood cell count, kidney dysfunction [serum creatinine>1.13 mg/dL (to convert to micromoles/L, multiply by 88.4) or blood urea nitrogen >22.4 mg/dL (to convert to mmol/L, multiply by 0.357], cardiac arrest, and cardiogenic shock).

Participating hospitals were surveyed on their level of care (tertiary or nontertiary), teaching status (college-affiliated teaching hospital, non–college-affiliated teaching hospital, or nonteaching hospital), and capacity to perform percutaneous coronary intervention. Hospital region was classified as Western, Central, or Eastern according to the China Census definitions.^14,17^

### Measurements of Process of Care and Outcomes

We evaluated the use of the following evidence-based treatments recommended by guidelines for AMI^2,3,4,5,6,7^: (1) reperfusion therapy, defined as primary percutaneous coronary intervention or fibrinolytic therapy in patients with ST-elevation myocardial infarction; (2) aspirin within 24 hours of admission; (3) clopidogrel within 24 hours of admission; (4) β-blockers within 24 hours of admission; (5) angiotensin-converting enzyme inhibitors (ACEIs) or angiotensin receptor blockers (ARBs) during hospitalization; and (6) statins during hospitalization. Use rates were calculated among patients considered ideal candidates for treatment (ie, patients who were clinically eligible and without contraindications; eAppendix in the Supplement). For each of the 6 treatments, we calculated the proportion of ideal patients who received that treatment. A composite use rate was calculated by dividing the number of times that a hospital successfully delivered each of the treatments to an ideal patient by the total number of opportunities that the hospital had to deliver such therapies.^18^

We measured 2 prespecified outcomes: in-hospital mortality, defined as any death or withdrawal from treatment because of terminal status during the hospitalization, and 5-day mortality, defined as any death or withdrawal from treatment because of terminal status within 5 days of admission, considering that difference in length of stay between periods could bias the comparison of in-hospital mortality. Because many patients in China are reluctant to die in hospital, treatment withdrawal due to terminal status is common and was included in our mortality outcomes.^19^ Cardiologists at the study coordinating center adjudicated the clinical status of patients who withdrew from treatment to confirm that the withdrawal was not for other reasons, such as financial issues. We also compared the length of stay, defined as the number of days between admission and discharge, to confirm the difference across regions.

### Statistical Analysis

Considering the health care reform was launched in 2009, we combined patients in the 2001, 2006, 2011, and 2015 data sets into 2 groups: 2001-2006 and 2011-2015. We conducted descriptive analysis to report percentages for categorical variables and medians (interquartile ranges [IQRs]) or means (SDs) for continuous variables. Patient characteristics, hospital characteristics, and outcomes were compared across regions using χ^2^ tests for categorical variables and Kruskal-Wallis tests for continuous or discrete variables.

We fit a mixed model with patient and hospital random intercepts to model the composite use of treatment measure as a function of patient demographic and clinical characteristics, study period (2011-2015, yes/no), region (Eastern, Central vs Western), and interaction terms between the study period and region variables. We repeated this model for the mortality outcomes. We fit an additional mixed model with hospital random intercepts to evaluate the association between treatment use, namely delivering all recommended treatments, and outcomes (in-hospital and 5-day mortality), adjusted for patient demographic and clinical characteristics. For age, the only involved variable with missing values, we imputed sample medians. All statistical tests were 2-sided, with a *P* value <.05 considered statistically significant. Statistical analysis was performed using SAS, version 9.4 (SAS Institute) from October 1 to October 31, 2019.

## Results

### Study Sample

At 153 hospitals across China, we sampled a total of 27046 hospitalizations for AMI. For 2001-2006, there were 1173 hospitalizations in the Western region, 1329 in the Central, and 4263 in the Eastern; for 2011-2015, there were 4403 hospitalizations in the Western region, 5227 in the Central, and 10651 in the Eastern. Of the participating hospitals, 45 were in Western China, 48 Central, and 60 Eastern. Hospital characteristics differed significantly across these geographic regions, with advanced cardiac care most readily available at Eastern hospitals, especially regarding the rate of catheterization laboratory (Western 40.0% [18 of 45], Central 37.5% [18 of 48], and Eastern 68.3% [41 of 60]; *P* = .002) (eTable 1 in the Supplement).

Most of overall rates of patient characteristics increased (eg, the rate of hypertension from 46.4% to 54.3% and the rate of diabetes from 15.0% to 20.4%) while some decreased (eg, the rate of left bundle branch block from 1.9% to 1.3%, and the rate of kidney dysfunction from 41.9% to 33.7%) (Table 1). Information about age was missing for 0 to 1% of cases. In both 2001-2006 and 2011-2015, the median (IQR) age was 66 (56-73) years. The Eastern region had persistently the highest proportion of women (Western 27.5%, Central 27.2%, and Eastern 31.1% in 2001-2006; *P* = .004; Western 29.6%, Central 29.5%, and Eastern 32.7% in 2011-2015; *P* < .001). Cardiovascular risk factors (eg, hypertension, diabetes, and dyslipidemia) and history of cardiovascular conditions were most prevalent among patients treated at Eastern hospitals during both study periods. There was also regional variation in clinical profiles at presentation.

**Table 1.** Patient Characteristics

Characteristic	Patients in 2001-2006, No. (%)				*P* value^a^	Patients in 2011-2015, No. (%)				*P* value^b^	*P* for interaction
	Overall (n = 6765)	Western (n = 1173)	Central (n = 1329)	Eastern (n = 4263)		Overall (n = 20 281)	Western (n = 4403)	Central (n = 5227)	Eastern (n = 10 651)		
**Demographic**											
Age (range), y	66 (56-73)	65 (55-73)	65 (54-73)	67 (56-74)	<.001	66 (56-73)	67 (56-75)	65 (56-75)	67 (57-76)	<.001	<.001
Female sex	2010 (29.7)	322 (27.5)	361 (27.2)	1327 (31.1)	.004	6328 (31.2)	1303 (29.6)	1543 (29.5)	3482 (32.7)	<.001	<.001
CVD risk factor											
Hypertension	3142 (46.4)	479 (40.8)	552 (41.5)	2111 (49.5)	<.001	11 019 (54.3)	2218 (50.4)	2618 (50.1)	6183 (58.1)	<.001	.59
Diabetes	1014 (15.0)	142 (12.1)	153 (11.5)	719 (16.9)	<.001	4136 (20.4)	763 (17.3)	865 (16.6)	2508 (23.6)	<.001	<.001
Dyslipidemia	249 (3.7)	42 (3.6)	28 (2.1)	179 (4.2)	.002	1450 (7.1)	242 (5.5)	286 (5.5)	922 (8.7)	<.001	<.001
Current smoker	2096 (31.0)	381 (32.5)	404 (30.4)	1311 (30.8)	.46	6735 (33.2)	1437 (32.6)	1649 (31.6)	3649 (34.3)	.002	<.001
**Medical history**											
Ischemic stroke	621 (9.2)	67 (5.7)	108 (8.1)	446 (10.5)	<.001	2364 (11.7)	428 (9.7)	527 (10.1)	1409 (13.2)	<.001	<.001
Coronary heart disease	1500 (22.2)	212 (18.1)	253 (19.0)	1035 (24.3)	<.001	5015 (24.7)	926 (21.0)	1288 (24.6)	2801 (26.3)	<.001	.004
Myocardial infarction	665 (9.8)	90 (7.7)	107 (8.1)	468 (11.0)	<.001	2034 (10.0)	389 (8.8)	453 (8.7)	1192 (11.2)	<.001	.54
**Clinical characteristic at presentation**											
No chest discomfort	559 (8.3)	92 (7.8)	120 (9.0)	347 (8.1)	.50	1961 (9.7)	502 (11.4)	439 (8.4)	1020 (9.6)	<.001	<.001
LBBB	128 (1.9)	29 (2.5)	33 (2.5)	66 (1.6)	.03	254 (1.3)	64 (1.5)	58 (1.1)	132 (1.2)	.31	<.001
Cardiac arrest	79 (1.2)	15 (1.3)	13 (1.0)	51 (1.2)	.75	260 (1.3)	52 (1.2)	55 (1.1)	153 (1.4)	.10	<.001
Cardiogenic shock	377 (5.6)	81 (6.9)	73 (5.5)	223 (5.2)	.09	1296 (6.4)	284 (6.5)	334 (6.4)	678 (6.4)	.98	<.001
Acute stroke	93 (1.4)	14 (1.2)	15 (1.1)	64 (1.5)	.50	508 (2.5)	121 (2.8)	152 (2.9)	235 (2.2)	.02	<.001
Kidney dysfunction^c^^,^^d^	2228 (41.9)	440 (45.1)	366 (41.5)	1422 (41.2)	.09	6159 (33.7)	1478 (36.5)	1604 (34.6)	3077 (32.1)	<.001	<.001
HR >90 beats/min	1620 (23.9)	275 (23.4)	326 (24.5)	1019 (23.9)	.81	4777 (23.6)	1052 (23.9)	1237 (23.7)	2488 (23.4)	.76	<.001
SBP <100 mm Hg	912 (13.5)	163 (13.9)	202 (15.2)	547 (12.8)	.08	1992 (9.8)	539 (12.2)	581 (11.1)	872 (8.2)	<.001	<.001
White blood cell count^d^											
6000-12 000/μL	3540 (64.3)	631 (63.5)	616 (61.1)	2293 (65.4)	.04	11 868 (64.6)	2583 (63.7)	2960 (63.0)	6325 (65.8)	.002	<.001
>12 000/μL	1384 (25.1)	262 (26.4)	263 (26.1)	859 (24.5)	.35	4342 (23.6)	1023 (25.2)	1145 (24.4)	2174 (22.6)	.002	<.001

Abbreviations: CVD indicates cardiovascular disease; HR, heart rate; LBBB, left bundle branch block; SBP, systolic blood pressure.

SI conversion factors: To convert white blood cell count to ×10^9^/L, multiply by 0.001.

^a^ Test for difference among the Western, Central, and Eastern regions in 2001-2006.

^b^ Test for difference among the Western, Central, and Eastern regions in 2011-2015.

^c^ Kidney dysfunction was defined as serum creatinine >1.13 mg/dL (to convert to micromoles per liter, multiply by 88.4) or blood urea nitrogen >22.4 mg/dL (to convert to mmol/L, multiply by 0.357).

^d^ Percentages were calculated among patients with available measurements.

### Geographic Variation and Temporal Change in Process of Care

**Figure 1.**
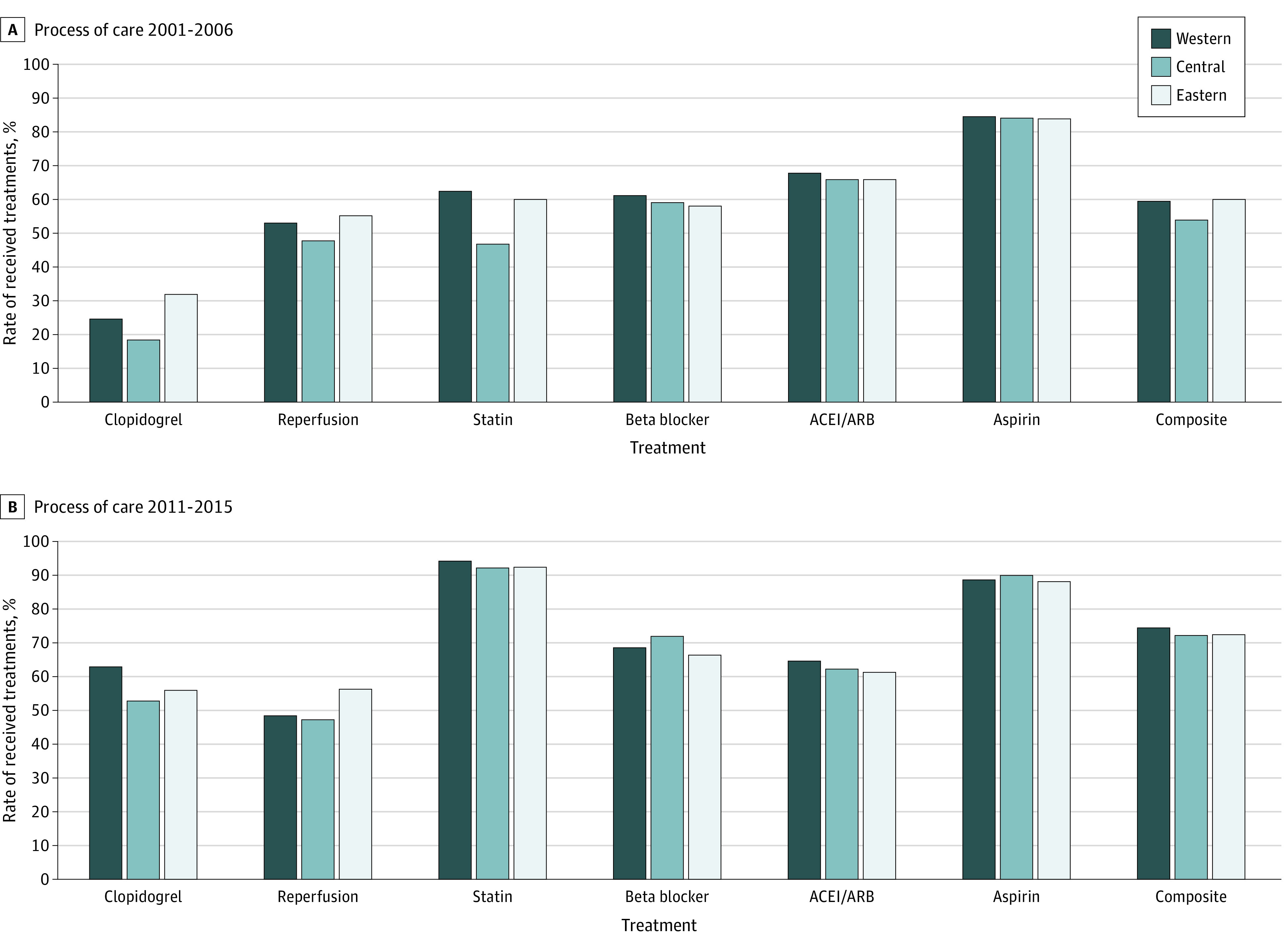
Process of Care Among Patients With Acute Myocardial Infarction A, Process of care in 2001-2006. B, Process of care in 2011-2015. Use of treatment was calculated among ideal patients (patients who were clinically eligible without contraindications). Reperfusion included primary coronary intervention or thrombolysis in patients with ST-elevation myocardial infarction. ACEI indicates angiotensin-converting enzyme inhibitor; ARB, angiotensin receptor blocker.

The composite use rate for guideline-recommended treatments varied significantly across the 3 regions in 2001-2006 (Western 59.4%, Central 53.9%, and Eastern 59.9%; *P* < .001) and 2011-2015 (Western 74.5%, Central 72.2%, and Eastern 72.5%; *P* < .001) (Figure 1; eTable 2 in the Supplement). There were also differences by region in the delivery of each individual treatment in 2011-2015 (eg, for clopidogrel, Western 62.8%, Central 52.9%, and Eastern 56.1%; *P* < .001). Variation in reperfusion tended to be widened from 2001-2006 to 2011-2015, for decrease in Western region and increase in Eastern region (Western from 53.0% to 48.6% and Eastern from 55.3% to 56.3%). The pattern of delivering aspirin across regions was similar. Performance was improved for both clopidogrel (from 28.1% in 2001-2006 to 56.7% in 2011-2015) and statins (from 57.8% to 92.7%) with the disparity between the highest and the lowest regions narrowed (for clopidogrel, highest 32.0% and lowest 18.4% in 2001-2006; 62.8% and 52.9% in 2011-2015; for statins, 62.4% and 46.9% in 2001-2006; 94.2% and 92.2% in 2011-2015). The variation in β-blockers (highest 61.2% and lowest 58.1% in 2001-2006; 71.9% and 66.6% in 2011-2015) and ACEIs/ARBs (67.8% and 65.9% in 2001-2006; 64.6% and 61.4% in 2011-2015) became more notable.

There was a significant difference across regions and the odds of treatment among ideal candidates was 17% lower comparing the lowest region (Central) with the highest region (Western) (odds ratio [OR], 0.83; 95% CI, 0.76-0.91; *P* < .001) (Table 2; eTable 3 in the Supplement). The variation across regions changed over time with variation between the 2 higher regions (Eastern and Western) narrowed (time-by-region interaction: OR, 0.83; 95% CI, 0.76-0.91; *P* < .001). In analyses stratified by region, the odds of hospitals providing recommended treatments increased by 110% for the Western region (OR, 2.10; 95% CI, 1.94-2.28; *P* < .001), by 115% for the Central region (2.15; 2.00-2.30; *P* < .001), and by 71% for the Eastern region (1.71; 1.64-1.79; *P* < .001) (eTable 4 in the Supplement).

**Table 2.** Change in Process of Care and Outcomes

Characteristics	Composite use of treatments		In-hospital mortality		Five-day mortality	
	OR (95% CI)	*P* value^a^	OR (95% CI)	*P* value^a^	OR (95% CI)	*P* value^a^
Years 2011-2015^b^	2.06 (1.91-2.23)	<.001	0.77 (0.63-0.95)	.01	0.80 (0.64-1.00)	.05
Region						
Eastern	1.02 (0.95-1.10)	<.001	0.80 (0.62-1.05)	.06	0.76 (0.57-1.00)	.04
Central	0.83 (0.76-0.91)		0.68 (0.50-0.93)		0.66 (0.47-0.92)	
Western	1 [Reference]		1 [Reference]		1 [Reference]	
Year × region						
2011-2015^b^ × Eastern	0.83 (0.76-0.91)	<.001	1.04 (0.82-1.32)	.81	1.01 (0.78-1.31)	.98
2011-2015^b^ × Central	1.05 (0.94-1.16)		0.97 (0.72-1.30)		0.98 (0.71-1.36)	
2011-2015^b^ × Western	1 [Reference]		1 [Reference]		1 [Reference]	

Abbreviations: OR, odds ratio.

^a^ Adjusted for patient characteristics, such as age, sex, current smoker, hypertension, diabetes, dyslipidemia, prior coronary heart disease, prior myocardial infarction, prior stroke, low systolic blood pressure, high heart rate, white blood cell count, kidney dysfunction, cardiac arrest, and cardiogenic shock.

^b^ Patients in the years 2001-2006 were the reference group.

### Geographic Variation and Temporal Change in Outcomes

**Figure 2.**
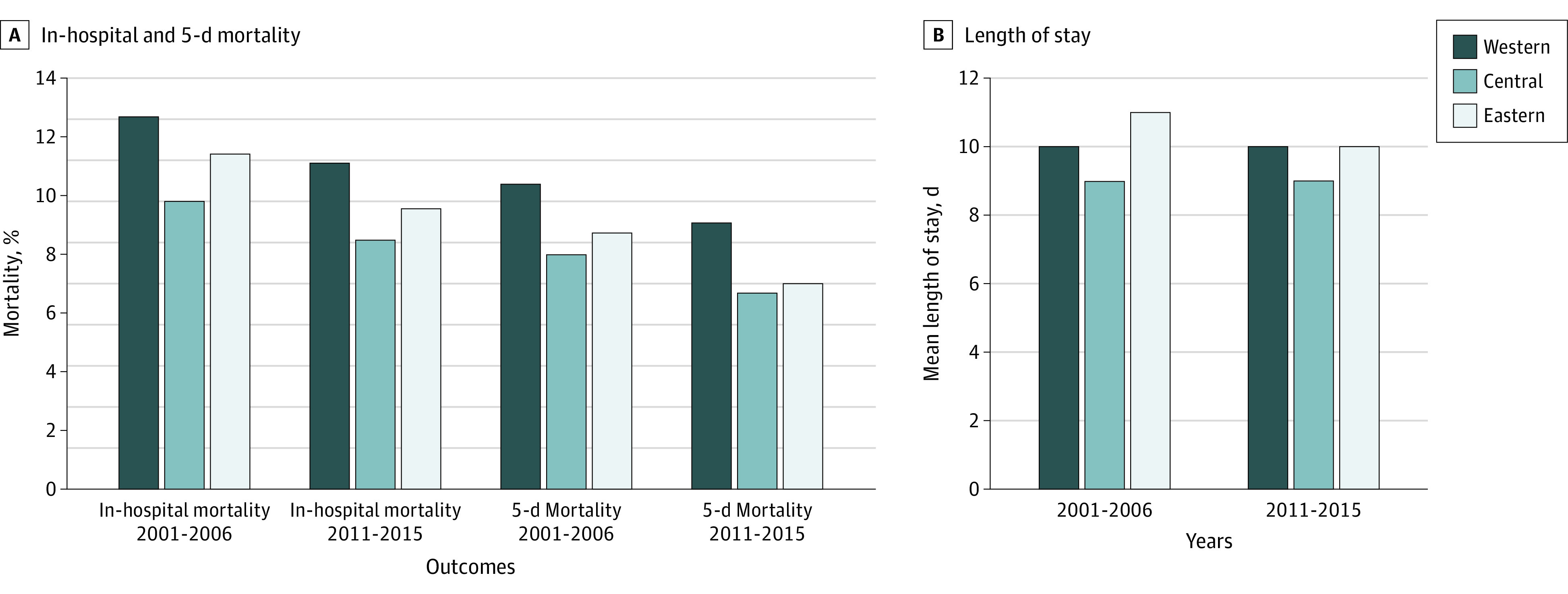
Outcomes Among Patients With Acute Myocardial Infarction A, In-hospital and 5-day mortality. B, Length of stay. In-hospital mortality and 5-day mortality were defined as any death or withdrawal from treatment because of terminal status during the hospitalization and within 5 days from the date of admission, respectively.

In-hospital mortality across regions had little difference in 2001-2006 (Western 12.7%, Central 9.8%, and Eastern 11.4%; *P* = .07) and varied significantly in 2011-2015 (11.1%, 8.5%, and 9.6%; *P* < .001) (Figure 2; eTable 2 in the Supplement). Similar results were found for 5-day mortality. The median (IQR) length of stay was 11 (6-16) days in 2001-2006 and 9 (6-14) in 2011-2015, shortest for patients in the Central hospitals in both periods (for 2001-2006, Western 10 [5-17] days, Central 9 [5-14] days, and Eastern 11 [6-17] days; *P* < .001; while for 2011-2015, Western 10 [5-14] days, Central 9 [5-13] days, and Eastern 10 [6-14] days; *P* < .001).

There is a borderline difference across regions in in-hospital mortality and the odds of in-hospital mortality were 1.46 times higher for patients in the highest region compared with those in the lowest region (95% CI, 1.07-2.00; *P* = .06). Regional patterns did not change, as indicated by the nonsignificant time-by-region interaction terms. For each region, the odds of in-hospital mortality decreased, by 22% for the Western region (OR, 0.78; 95% CI, 0.63-0.96; *P* = .02), by 28% for the Central region (0.72; 0.58-0.90; *P* = .004), and by 19% for the Eastern region (0.81; 0.72-0.91; *P* = .001; eTable 6 in the Supplement).

There was a significant variation across regions in 5-day mortality and the odds were 1.52 times higher for patients in the highest region compared with those in the lowest region (95% CI, 1.09-2.11; *P* = .04) (Table 2; eTable 7 in the Supplement). There was no significant interaction between region and time period. For the Western and Eastern regions, the odds of 5-day mortality decreased significantly between 2001-2005 and 2011-2015 (eTable 8 in the Supplement).

### Association Between Process of Care and Outcomes

Delivering all recommended treatments to an ideal patient was associated with decreased odds of in-hospital mortality by 38% to 51% for each region (Western, OR, 0.50, 95% CI, 0.37-0.67; *P* < .001; Central, OR, 0.49, 95% CI, 0.35-0.69; *P* < .001; Eastern, OR, 0.62, 95% CI, 0.52-0.74; *P* < .001) (Table 3; eTable 9 in the Supplement). Similar patterns were found for 5-day mortality, with a 43% to 73% reduction in odds for different regions (Western, OR, 0.41, 95% CI, 0.28-0.59; *P* < .001; Central, OR, 0.27, 95% CI, 0.16-0.45; *P* < .001; Eastern, OR, 0.57, 95% CI, 0.45-0.73; *P* < .001) (eTable 10 in the Supplement).

**Table 3.** Association Between Process of Care and Outcomes Specified by Regions

Outcomes	Characteristics	Western		Central		Eastern	
		OR (95% CI)	*P* value^a^	OR (95% CI)	*P* value^a^	OR (95% CI)	*P* value^a^
In-hospital mortality	Receiving all treatments^b^	0.50 (0.37-0.67)	<.001	0.49 (0.35-0.69)	<.001	0.62 (0.52-0.74)	<.001
5-d Mortality	Receiving all treatments^b^	0.41 (0.28-0.59)	<.001	0.27 (0.16-0.45)	<.001	0.57 (0.45-0.73)	<.001

Abbreviations: OR, odds ratio.

^a^ Adjusted for patient characteristics, such as age, sex, current smoker, hypertension, diabetes, dyslipidemia, prior coronary heart disease, prior myocardial infarction, prior stroke, low systolic blood pressure, high heart rate, white blood cell count, kidney dysfunction, cardiac arrest, and cardiogenic shock.

^b^ Use of treatment determined among ideal patients (patients who were clinically eligible without contraindications).

## Discussion

In this nationally representative investigation of AMI in China, we found a significant geographic variation in process of care and in-hospital outcomes. The variation across regions changed in treatments for ideal candidates while that between the 2 higher regions narrowed. The in-hospital mortality and 5-day mortality varied across regions and the regional variation did not change from 2001-2006 to 2011-2015.

We expanded on previous work^19,20^ by incorporating 2015 data and investigating geographic variation over more than a decade to assess the national efforts to improve the quality and equity of care. Repeated measures in the same series of randomly selected hospitals reflected temporal change of variations with little bias. In the process of achieving common prosperity, development of China is fairly unbalanced by geographic areas with lagging economy and weaker health infrastructure from Eastern compared with Western region.^14^ In the present study, we observed significant differences in the use of guideline-recommended treatments across China, with hospitals in the Western region having the best performance, particularly for clopidogrel, ACEIs/ARBs, and statins. The Western region is the least economically developed region in China,^21^ further distinguishing the performance and progress made by the hospitals in this region. This finding may be partly attributed to the Western Development Program,^22^ which was initiated by the Chinese government in 2000 to support health care and economic development in the Western region. From 2001 to 2009, 86.3 billion RMB (approximately 12.8 billion US dollars) was allocated to public health in the Western region, accounting for 46.7% of the country’s public health funds.^15^ A study based on national statistical data showed that the Western region provided relatively more health care services with low resources allocation from 2009 to 2013,^23^ providing evidence of the region’s continued achievement in the provision of health services. Because of the variation in treatment patterns across regions, further monitoring of process of care measures and establishment of incentive mechanisms are warranted to ensure continued improvement in quality of care.

Hospitals in the Central region had better mortality outcomes with relatively poorer performance of care delivery. One possible explanation is that patients in the Central region may have already had lower risk of poor outcomes at presentation. As shown in Table 1, diabetes and kidney dysfunction were less prevalent in the Central region. Even though we used multivariable models to control the case-mix in outcome comparisons, residual confounding could occur in such an observational study, as some clinical conditions may not be fully reflected by the data collected from medical records retrospectively. Additionally, there were fewer ideal patients for treatments in aggregate in the Central region than in other regions (eTable 11 in the Supplement). That might also diminish the effect of treatments on outcomes in the overall population.

Although delivery rates for guideline-recommended treatments were better in the Western region when compared with other regions, in-hospital and 5-day mortality were both higher. On the one hand, fewer hospitals in the Western region had cardiac catheterization laboratories, which made primary percutaneous coronary intervention more difficult to achieve. The between-period decrease in reperfusion rates in the Western and increase in the Eastern might associate with the changing differences in mortality between regions. On the other hand, some quality indicator affecting outcomes, like the timeliness of reperfusion, were not included in our measurements, which could be another reason for the disparity between treatments and outcomes we observed.

Despite geographic variation, use of guideline-recommended treatments increased and the care variation decreased in some ways. Over the past decades, there have been several national health campaigns involving management of AMI. The national health care reform launched in 2009,^13^ focused on 5 major fields including promotion of public health service and equity. Additionally, a nationwide program was initiated in 2011 that emphasized the construction of a management system and regional first aid network as well as training to standardize AMI process of care.^24^ Consequently, access to medical services and insurance protection has improved dramatically during the past decade across geographic regions.^14,25^ Our findings are generally consistent with those of previous studies^14,19,20^ that support the benefits of these campaigns. However, care delivery remains suboptimal and disparities remained across China when compared with that in the United States and United Kingdom where reperfusion therapy, β-blockers, and ACEIs/ARBs are used at much higher rates.^26,27^ Additional measures should be taken to further narrow regional care disparity across the country.

### Limitations

Our study has limitations. First, the patient characteristics, treatments and outcomes included were obtained from medical records retrospectively, and there may be undocumented information, which causes lack of some quality indicators (for example time to reperfusion that influenced the outcomes), and residual confounding in case-mix adjustment. However, the potential bias should be small. Second, shorter length of stay may have biased the study toward lower in-hospital event rates. Therefore, we conducted an analysis using a uniform 5-day timeframe for mortality and found similar results. Third, our study was unable to assess outcomes after discharge and therefore cannot provide evidence of geographic variation in outcomes after patients leave the hospital. We focused on in-hospital outcomes, which are easily measured, reliable, and more likely to be influenced by in-hospital vs postdischarge care. Additionally, mortality outcomes included withdrawal from treatment because of terminal status. However, it was adjudicated at the coordinating center, blinded as to year and/or region, to avoid bias.

## Conclusions

In this cross-sectional study, geographic variation occurred in the process of care and outcomes in China. To achieve equitable care and optimal outcomes for patients with AMI, more investigations into the underlying mechanisms and targeted efforts to reinforce treatment standards are warranted in combination with systematic quality evaluation and incentive mechanisms.

## Notes

Supplement. **eAppendix.** Ideal Candidates for the Treatments **eTable 1.** Hospital Characteristics by Regions **eTable 2.** Process of Care and Outcomes Among Patients with Acute Myocardial Infarction **eTable 3.** Composite Utilization of Treatments Model **eTable 4.** Composite Utilization of Treatments Models for Different Regions **eTable 5.** In-hospital Mortality Model **eTable 6.** In-hospital Mortality Models for Different Regions **eTable 7.** Five-day Mortality Model **eTable 8.** Five-day Mortality Models for Different Regions **eTable 9.** Association Between Utilization of All Treatments and In-hospital Mortality in Different Regions **eTable 10.** Association Between Utilization of All Treatments and 5-day Mortality in Different Regions **eTable 11.** Ideal Candidates for Treatments among Patients with Acute Myocardial Infarction
