# Supplementary material for: Geographic Variation in Process and Outcomes of Care for Patients With Acute Myocardial Infarction in China From 2001 to 2015
Source: JAMA Netw Open. 2020 Oct 23;3(10):e2021182. doi: 10.1001/jamanetworkopen.2020.21182 (PMC7584924; doi:10.1001/jamanetworkopen.2020.21182)
Supplement: Supplement. — eAppendix. Ideal Candidates for the Treatments eTable 1. Hospital Characteristics by Regions eTable 2. Process of Care and Outcomes Among Patients with Acute Myocardial Infarction eTable 3. Composite Utilization of Treatments Model eTable 4. Composite Utilization of Treatments Models for Different Regions eTable 5. In-hospital Mortality Model eTable 6. In-hospital Mortality Models for Different Regions eTable 7. Five-day Mortality Model eTable 8. Five-day Mortality Models for Different Regions eTable 9. Association Between Utilization of All Treatments and In-hospital Mortality in Different Regions eTable 10. Association Between Utilization of All Treatments and 5-day Mortality in Different Regions eTable 11. Ideal Candidates for Treatments among Patients with Acute Myocardial Infarction [file jamanetwopen-e2021182-s001.pdf]

Supplemental Online Content

Zhong Q, Gao y, Zheng X, et al. Geographic variation in process and outcomes of care for patients with acute myocardial infarction in China from 2001 to 2015. *JAMA Netw Open*. 2020;3(10):e2021182. doi:10.1001/jamanetworkopen.2020.21182

eAppendix. Ideal Candidates for the Treatments
eTable 1. Hospital Characteristics by Regions
eTable 2. Process of Care and Outcomes Among Patients with Acute Myocardial Infarction
eTable 3. Composite Utilization of Treatments Model
eTable 4. Composite Utilization of Treatments Models for Different Regions
eTable 5. In-hospital Mortality Model
eTable 6. In-hospital Mortality Models for Different Regions
eTable 7. Five-day Mortality Model
eTable 8. Five-day Mortality Models for Different Regions
eTable 9. Association Between Utilization of All Treatments and In-hospital Mortality in Different Regions
eTable 10. Association Between Utilization of All Treatments and 5-day Mortality in Different Regions
eTable 11. Ideal Candidates for Treatments among Patients with Acute Myocardial Infarction

This supplemental material has been provided by the authors to give readers additional information about their work.

© 2020 Zhong Q et al. *JAMA Network Open*.

eAppendix. Ideal Candidates for the Treatments

For reperfusion therapy, we included patients who were admitted within 12 hours of symptom onset and did not receive reperfusion therapy before hospital presentation. We excluded patients with any contraindications (history of hemorrhagic stroke, active bleeding at presentation, or any other physician documented contraindications for fibrinolytic therapy if the patient was treated in a non-percutaneous coronary intervention [PCI]-capable hospital; allergy to contrast agents or any other documented contraindication to PCI if the patient was treated in a PCI-capable hospital).

For aspirin, we excluded patients with any contraindications for aspirin (allergy to aspirin, active bleeding on admission, history of hemorrhagic stroke, or other documented contraindications).

For clopidogrel, we excluded patients who participated in the ClOpidogrel and Metoprolol in Myocardial Infarction Trial (COMMIT) and patients with any contraindications for clopidogrel (allergy to clopidogrel, active bleeding on admission, history of hemorrhagic stroke, or other documented contraindications).

For beta-blockers, we excluded patients who participated in the ClOpidogrel and Metoprolol in Myocardial Infarction Trial (COMMIT) and patients with any contraindications for beta-blockers (allergy to beta-blockers, cardiogenic shock on admission, heart failure on admission, second- or third-degree atrioventricular block with no pacemaker implanted, systolic blood pressure <100 mmHg on admission, bradycardia [heart rate <60 beats/min] on admission without taking a beta-blocker, or other documented contraindications).

For angiotensin-converting enzyme inhibitors (ACEIs) or angiotensin receptor blockers (ARBs), we excluded patients with any contraindications for ACEIs (allergy to ACEIs, hyperkalemia [serum potassium >5.5 mmol/L during hospitalization], creatinine >265 μmol/L during hospitalization, pregnancy or breast feeding, or other documented contraindications).

For statins, we excluded patients with any contraindications for statins (allergy to statins).

© 2020 Zhong Q et al. *JAMA Network Open*.

eTable 1. Hospital Characteristics by Region

Hospital Characteristics, n (%)	Western (n=45)	Central (n=48)	Eastern (n=60)	P Value
Level of hospital care[a]				0.004
Tertiary	18 (40.0)	12 (25.0)	34 (56.7)	
Secondary or lower	27 (60.0)	36 (75.0)	26 (43.3)	
Type of hospital				<0.001
Medical college affiliated	8 (17.8)	7 (14.6)	27 (45.0)	
Teaching, but not medical college affiliated	15 (33.3)	16 (33.3)	22 (36.7)	
Non-teaching	22 (48.9)	25 (52.1)	11 (18.3)	
CCU in hospital in 2011	23 (51.1)	20 (41.7)	46 (76.7)	0.001
Catheterization laboratory in hospital in 2011	18 (40.0)	18 (37.5)	41 (68.3)	0.002

CCU indicates coronary care unit.

[a] Hospitals in China are classified into three levels according to the standard of assessment established by the national health commission, based on hospital facilities and clinical capacities. Tertiary is the highest level.

© 2020 Zhong Q et al. *JAMA Network Open*.

eTable 2. Process of Care and Outcomes Among Patients with Acute Myocardial Infarction

	2001-2006				P Value[a]	2011-2015				P Value[b]
	Overall (n=6765)	Western (n=1173)	Central (n=1329)	Eastern (n=4263)		Overall (n=20281)	Western (n=4403)	Central (n=5227)	Eastern (n=10651)	
**Process of Care[c]**										
Reperfusion[d], n (%)	1395 (53.5)	238 (53.0)	225 (47.8)	932 (55.3)	0.02	3460 (52.3)	730 (48.6)	790 (47.4)	1940 (56.3)	<0.001
Aspirin, n (%)	5112 (84.0)	887 (84.6)	992 (84.1)	3233 (83.9)	0.86	15465 (88.8)	3340 (88.6)	3996 (90.1)	8129 (88.2)	0.001
Clopidogrel, n (%)	1675 (28.1)	259 (24.8)	214 (18.4)	1202 (32.0)	<0.001	9919 (56.7)	2380 (62.8)	2352 (52.9)	5187 (56.1)	<0.001
β-blocker, n (%)	1596 (58.9)	305 (61.2)	312 (59.1)	979 (58.1)	0.45	5567 (68.3)	1243 (68.7)	1436 (71.9)	2888 (66.6)	<0.001
ACEI/ARB, n (%)	3988 (66.2)	702 (67.8)	778 (65.9)	2508 (65.9)	0.49	10611 (62.3)	2385 (64.6)	2701 (62.3)	5525 (61.4)	0.003
Statin, n (%)	3595 (57.8)	668 (62.4)	564 (46.9)	2363 (59.9)	<0.001	16599 (92.7)	3655 (94.2)	4182 (92.2)	8762 (92.3)	<0.001
Composite, n (%)	17361 (58.6)	3059 (59.4)	3085 (53.9)	11217 (59.9)	<0.001	61621 (72.8)	13733 (74.5)	15457 (72.2)	32431 (72.5)	<0.001
**Outcomes**										
In-hospital mortality, n (%)	766 (11.3)	149 (12.7)	130 (9.8)	487 (11.4)	0.07	1950 (9.6)	489 (11.1)	443 (8.5)	1018 (9.6)	<0.001
5-day mortality, n (%)	600 (8.9)	122 (10.4)	106 (8.0)	372 (8.7)	0.09	1499 (7.4)	400 (9.1)	349 (6.7)	750 (7.0)	<0.001
LOS, median (IQR), d	11 (6-16)	10 (5-17)	9 (5-14)	11 (6-17)	<0.001	9 (6-14)	10 (5-14)	9 (5-13)	10 (6-14)	<0.001

ACEI indicates angiotensin-converting enzyme inhibitor; ARB, angiotensin receptor blocker; IQR, interquartile range; LOS, length of stay.

[a] Test for difference among the Western, Central, and Eastern regions in 2001-2006.

[b] Test for difference among the Western, Central, and Eastern regions in 2011-2015.

[c] Use of treatment was calculated among ideal patients.

[d] Reperfusion rate was calculated among patients with ST-elevation myocardial infarction.

© 2020 Zhong Q et al. *JAMA Network Open*.

eTable 3. Composite Utilization of Treatments Model

Characteristics	OR	95% CI	P value
Age, every 5 years	0.97	0.97-0.98	<0.001
Female	0.92	0.89-0.95	<0.001
Hypertension	1.45	1.41-1.50	<0.001
Diabetes	1.14	1.10-1.19	<0.001
Dyslipidemia	1.20	1.13-1.28	<0.001
Current smoker	1.20	1.16-1.24	<0.001
History of CHD	0.91	0.87-0.95	<0.001
Prior ischemic stroke	0.92	0.88-0.97	0.001
Prior myocardial infarction	1.10	1.03-1.17	0.003
No chest discomfort	0.60	0.57-0.64	<0.001
Renal dysfunction	0.95	0.92-0.99	0.008
LBBB	1.06	0.93-1.21	0.36
Cardiac arrest	0.92	0.79-1.07	0.26
Cardiogenic shock	0.96	0.89-1.04	0.30
Acute stroke	0.63	0.57-0.70	<0.001
Heart rate >90 beats/min	0.91	0.88-0.95	<0.001
SBP <100 mm Hg	0.79	0.75-0.84	<0.001
White blood cell count			
6000/µL-12 000/µL	1.20	1.16-1.25	<0.001
>12 000/µL	1.21	1.15-1.26	<0.001
Years 2011-2015[a]	2.06	1.91-2.23	<0.001
Region			<0.001
Eastern	1.02	0.95-1.10	
Central	0.83	0.76-0.91	
Western(Reference)	1	-	
Year*regeion			<0.001
2011-2015[a] × Eastern	0.83	0.76-0.91	
2011-2015[a] × Central	1.05	0.94-1.16	
2011-2015[a] ×Western(Reference)	1	-	

CHD indicates coronary heart disease; CI, confidence interval; LBBB, left bundle branch block; OR, odds ratio; SBP, systolic blood pressure.

[a] Patients in the years 2001-2006 were the reference group.

© 2020 Zhong Q et al. *JAMA Network Open*.

eTable 4. Composite Utilization of Treatments Models for Different Regions

Characteristics	Western			Central			Eastern		
	OR	95% CI	P value	OR	95% CI	P value	OR	95% CI	P value
Age, every 5 years	0.95	0.94-0.97	<0.001	0.97	0.95-0.98	<0.001	0.98	0.97-0.99	<0.001
Female	0.97	0.90-1.06	0.51	0.92	0.85-0.99	0.02	0.91	0.86-0.95	<0.001
Hypertension	1.36	1.26-1.46	<0.001	1.42	1.34-1.51	<0.001	1.5	1.44-1.57	<0.001
Diabetes	1.13	1.03-1.25	0.01	1.12	1.03-1.22	0.01	1.15	1.09-1.21	<0.001
Dyslipidemia	1.19	1.02-1.40	0.03	1.12	0.97-1.29	0.13	1.23	1.13-1.33	<0.001
Current smoker	1.34	1.24-1.45	<0.001	1.17	1.09-1.26	<0.001	1.16	1.11-1.22	<0.001
History of CHD	0.97	0.87-1.08	0.59	0.95	0.87-1.03	0.23	0.87	0.82-0.92	<0.001
Prior ischemic stroke	0.99	0.88-1.12	0.89	0.97	0.87-1.07	0.51	0.88	0.83-0.94	<0.001
Prior myocardial infarction	1.03	0.89-1.20	0.69	1.04	0.92-1.18	0.56	1.14	1.05-1.23	0.002
No chest discomfort	0.62	0.56-0.70	<0.001	0.59	0.53-0.66	<0.001	0.6	0.56-0.65	<0.001
Renal dysfunction	0.97	0.90-1.04	0.38	0.91	0.85-0.98	0.01	0.97	0.93-1.02	0.23
LBBB	1.25	0.95-1.64	0.11	0.99	0.76-1.29	0.94	1.01	0.84-1.21	0.91
Cardiac arrest	0.76	0.53-1.08	0.13	0.80	0.58-1.11	0.18	1.03	0.84-1.25	0.80
Cardiogenic shock	1.02	0.86-1.21	0.83	0.99	0.85-1.15	0.86	0.93	0.84-1.03	0.17
Acute stroke	0.58	0.46-0.72	<0.001	0.82	0.68-0.99	0.04	0.58	0.50-0.67	<0.001
Heart rate >90 beats/min	0.87	0.80-0.94	0.001	0.91	0.85-0.98	0.02	0.93	0.89-0.98	0.005
SBP <100 mm Hg	0.79	0.70-0.89	<0.001	0.80	0.72-0.89	<0.001	0.78	0.72-0.85	<0.001
White blood cell count									
6000/μL-12 000/μL	1.18	1.08-1.30	<0.001	1.18	1.10-1.27	<0.001	1.22	1.16-1.28	<0.001
>12 000/μL	1.16	1.04-1.29	0.01	1.2	1.10-1.32	<0.001	1.23	1.15-1.31	<0.001
Years 2011-2015[a]	2.10	1.94-2.28	<0.001	2.15	2.00-2.30	<0.001	1.71	1.64-1.79	<0.001

CHD indicates coronary heart disease; CI, confidence interval; LBBB, left bundle branch block; OR, odds ratio; SBP, systolic blood pressure.

[a] Patients in the years 2001-2006 were the reference group.

© 2020 Zhong Q et al. *JAMA Network Open*.

eTable 5. In-hospital Mortality Model

Characteristics	OR	95% CI	P value
Age, every 5 years	1.23	1.20-1.25	<0.001
Female	1.29	1.18-1.41	<0.001
Hypertension	0.88	0.81-0.96	0.004
Diabetes	1.28	1.16-1.42	<0.001
Dyslipidemia	0.77	0.63-0.94	0.01
Current smoker	0.64	0.57-0.71	<0.001
History of CHD	0.99	0.88-1.11	0.89
Prior ischemic stroke	1.32	1.18-1.49	<0.001
Prior myocardial infarction	0.94	0.80-1.10	0.44
No chest discomfort	1.35	1.20-1.52	<0.001
Renal dysfunction	1.70	1.56-1.86	<0.001
LBBB	1.18	0.90-1.55	0.24
Cardiac arrest	2.47	1.89-3.23	<0.001
Cardiogenic shock	3.12	2.75-3.54	<0.001
Acute stroke	2.24	1.84-2.74	<0.001
Heart rate >90 beats/min	1.78	1.63-1.94	<0.001
SBP <100 mm Hg	2.44	2.18-2.73	<0.001
White blood cell count			
6000/μL-12 000/μL	0.65	0.58-0.72	<0.001
>12 000/μL	1.34	1.19-1.50	<0.001
Years 2011-2015[a]	0.77	0.63-0.95	0.01
Region			0.059
Eastern	0.80	0.62-1.05	
Central	0.68	0.50-0.93	
Western(Reference)	1	-	
Year*region			0.805
2011-2015[a] × Eastern	1.04	0.82-1.32	
2011-2015[a] × Central	0.97	0.72-1.30	
2011-2015[a] x Western(Reference)	1	-	

CHD indicates coronary heart disease; CI, confidence interval; LBBB, left bundle branch block; OR, odds ratio; SBP, systolic blood pressure.

[a] Patients in the years 2001-2006 were the reference group.

© 2020 Zhong Q et al. *JAMA Network Open*.

eTable 6. In-hospital Mortality Models for Different Regions

Characteristics	Western			Central			Eastern		
	OR	95% CI	P value	OR	95% CI	P value	OR	95% CI	P value
Age, every 5 years	1.18	1.13-1.23	<0.001	1.24	1.19-1.30	<0.001	1.24	1.21-1.28	<0.001
Female	1.45	1.20-1.76	<0.001	1.19	0.97-1.45	0.10	1.28	1.13-1.43	<0.001
Hypertension	0.89	0.74-1.07	0.20	0.94	0.78-1.14	0.52	0.85	0.75-0.95	0.005
Diabetes	1.32	1.06-1.65	0.01	1.50	1.20-1.89	<0.001	1.21	1.06-1.38	0.005
Dyslipidemia	0.74	0.48-1.15	0.18	0.69	0.42-1.13	0.14	0.79	0.61-1.01	0.06
Current smoker	0.62	0.49-0.79	<0.001	0.67	0.52-0.86	0.002	0.63	0.54-0.73	<0.001
History of CHD	1.05	0.80-1.37	0.73	0.89	0.69-1.14	0.34	1.02	0.88-1.19	0.76
Prior ischemic stroke	1.39	1.05-1.83	0.02	1.37	1.05-1.78	0.02	1.30	1.12-1.51	<0.001
Prior myocardial infarction	1.02	0.70-1.48	0.92	0.80	0.53-1.18	0.26	0.94	0.76-1.15	0.54
No chest discomfort	1.29	1.02-1.65	0.04	1.61	1.25-2.08	<0.001	1.28	1.09-1.50	0.002
Renal dysfunction	1.72	1.43-2.08	<0.001	1.63	1.34-1.98	<0.001	1.73	1.54-1.95	<0.001
LBBB	1.26	0.73-2.17	0.40	1.85	1.07-3.17	0.03	0.92	0.62-1.37	0.69
Cardiac arrest	1.36	0.71-2.59	0.35	3.97	2.25-7.00	<0.001	2.52	1.78-3.57	<0.001
Cardiogenic shock	3.15	2.39-4.17	<0.001	3.40	2.60-4.46	<0.001	3.03	2.56-3.59	<0.001
Acute stroke	2.32	1.52-3.53	<0.001	1.84	1.22-2.79	0.004	2.41	1.83-3.17	<0.001
Heart rate >90 beats/min	1.70	1.41-2.04	<0.001	1.93	1.60-2.33	<0.001	1.76	1.57-1.98	<0.001
SBP <100 mm Hg	2.22	1.75-2.80	<0.001	2.62	2.06-3.33	<0.001	2.43	2.09-2.84	<0.001
White blood cell count									
6000/μL-12 000/μL	0.65	0.51-0.82	<0.001	0.76	0.60-0.96	0.02	0.62	0.54-0.72	<0.001
>12 000/μL	1.35	1.05-1.74	0.02	1.42	1.10-1.83	0.01	1.31	1.12-1.53	0.001
Years 2011-2015[a]	0.78	0.63-0.96	0.02	0.72	0.58-0.90	0.004	0.81	0.72-0.91	0.001

CHD indicates coronary heart disease; CI, confidence interval; LBBB, left bundle branch block; OR, odds ratio; SBP, systolic blood pressure.

[a] Patients in the years 2001-2006 were the reference group.

© 2020 Zhong Q et al. *JAMA Network Open*.

eTable 7. Five-day Mortality Model

Characteristics	OR	95% CI	P value
Age, every 5 years	1.20	1.17-1.23	<0.001
Female	1.34	1.21-1.48	<0.001
Hypertension	0.83	0.75-0.91	<0.001
Diabetes	1.21	1.08-1.36	0.001
Dyslipidemia	0.71	0.56-0.90	0.004
Current smoker	0.61	0.54-0.70	<0.001
History of CHD	0.92	0.81-1.05	0.22
Prior ischemic stroke	1.18	1.03-1.35	0.02
Prior myocardial infarction	0.89	0.73-1.07	0.22
No chest discomfort	1.23	1.08-1.40	0.002
Renal dysfunction	1.50	1.36-1.65	<0.001
LBBB	1.02	0.74-1.39	0.91
Cardiac arrest	2.38	1.79-3.15	<0.001
Cardiogenic shock	3.38	2.96-3.86	<0.001
Acute stroke	2.11	1.69-2.63	<0.001
Heart rate >90 beats/min	1.81	1.64-1.99	<0.001
SBP <100 mm Hg	2.85	2.53-3.22	<0.001
White blood cell count			
6000/μL-12 000/μL	0.58	0.52-0.65	<0.001
>12 000/μL	1.15	1.01-1.31	0.03
Years 2011-2015[a]	0.80	0.64-1.00	0.05
Region			0.040
Eastern	0.76	0.57-1.00	
Central	0.66	0.47-0.92	
Western(Reference)	1	-	
Year*region			0.983
2011-2015[a] × Eastern	1.01	0.78-1.31	
2011-2015[a] × Central	0.98	0.71-1.36	
2011-2015[a] x Western(Reference)	1	-	

CHD indicates coronary heart disease; CI, confidence interval; LBBB, left bundle branch block; OR, odds ratio; SBP, systolic blood pressure.

[a] Patients in the years 2001-2006 were the reference group.

© 2020 Zhong Q et al. *JAMA Network Open*.

eTable 8. Five-day Mortality Models for Different Regions

Characteristics	Western			Central			Eastern		
	OR	95% CI	P value	OR	95% CI	P value	OR	95% CI	P value
Age, every 5 years	1.17	1.12-1.23	<0.001	1.20	1.14-1.26	<0.001	1.21	1.18-1.25	<0.001
Female	1.51	1.23-1.86	<0.001	1.23	0.99-1.53	0.068	1.32	1.16-1.51	<0.001
Hypertension	0.82	0.67-1.01	0.06	0.84	0.68-1.04	0.106	0.82	0.72-0.94	0.004
Diabetes	1.41	1.10-1.79	0.01	1.37	1.06-1.77	0.016	1.10	0.94-1.28	0.23
Dyslipidemia	0.63	0.37-1.07	0.09	0.71	0.40-1.25	0.231	0.72	0.53-0.98	0.04
Current smoker	0.55	0.42-0.71	<0.001	0.64	0.48-0.84	0.001	0.64	0.54-0.76	<0.001
History of CHD	0.92	0.68-1.25	0.60	0.84	0.63-1.11	0.219	0.96	0.81-1.14	0.64
Prior ischemic stroke	1.20	0.88-1.64	0.26	1.26	0.94-1.69	0.125	1.15	0.96-1.37	0.12
Prior myocardial infarction	0.93	0.61-1.44	0.76	0.75	0.48-1.20	0.230	0.90	0.71-1.15	0.40
No chest discomfort	1.15	0.88-1.50	0.31	1.56	1.18-2.06	0.002	1.16	0.96-1.39	0.12
Renal dysfunction	1.51	1.23-1.86	<0.001	1.47	1.18-1.82	<0.001	1.52	1.33-1.75	<0.001
LBBB	0.79	0.40-1.54	0.48	1.95	1.10-3.47	0.023	0.82	0.52-1.30	0.40
Cardiac arrest	1.35	0.69-2.66	0.39	2.72	1.47-5.02	0.001	2.70	1.88-3.88	<0.001
Cardiogenic shock	3.63	2.71-4.85	<0.001	3.76	2.85-4.96	<0.001	3.17	2.65-3.80	<0.001
Acute stroke	2.84	1.82-4.42	<0.001	1.71	1.08-2.71	0.023	1.97	1.44-2.70	<0.001
Heart rate >90 beats/min	1.67	1.37-2.05	<0.001	2.04	1.66-2.50	<0.001	1.79	1.57-2.05	<0.001
SBP <100 mm Hg	2.39	1.86-3.07	<0.001	2.94	2.28-3.78	<0.001	3.01	2.56-3.54	<0.001
White blood cell count									
6000/μL-12 000/μL	0.68	0.52-0.88	0.003	0.64	0.49-0.82	0.001	0.52	0.45-0.61	<0.001
>12 000/μL	1.27	0.96-1.68	0.09	1.16	0.88-1.52	0.290	1.10	0.93-1.31	0.28
Years 2011-2015[a]	0.78	0.62-0.99	0.04	0.78	0.61-1.00	0.051	0.82	0.72-0.94	0.01

CHD indicates coronary heart disease; CI, confidence interval; LBBB, left bundle branch block; OR, odds ratio; SBP, systolic blood pressure.

[a] Patients in the years 2001-2006 were the reference group.

© 2020 Zhong Q et al. *JAMA Network Open*.

eTable 9. Association Between Utilization of All Treatments and In-hospital Mortality in Different Regions

Characteristics	In-hospital Mortality								
	Western			Central			Eastern		
	OR	95% CI	P value	OR	95% CI	P value	OR	95% CI	P value
Age, every 5 years	1.20	1.14-1.27	<0.001	1.27	1.20-1.34	<0.001	1.26	1.22-1.30	<0.001
Female	1.53	1.21-1.94	<0.001	1.36	1.06-1.75	0.02	1.30	1.13-1.51	<0.001
Hypertension	0.97	0.77-1.22	0.79	1.01	0.80-1.28	0.93	0.85	0.73-0.98	0.02
Diabetes	1.29	0.98-1.69	0.07	1.74	1.32-2.31	<0.001	1.26	1.08-1.47	0.004
Dyslipidemia	0.97	0.60-1.57	0.90	0.60	0.33-1.08	0.09	0.93	0.71-1.22	0.61
Current smoker	0.87	0.66-1.14	0.31	0.83	0.60-1.14	0.24	0.80	0.67-0.96	0.02
History of CHD	1.13	0.82-1.55	0.46	1.00	0.74-1.35	0.99	1.24	1.04-1.47	0.02
Prior ischemic stroke	1.59	1.16-2.17	0.004	1.35	0.98-1.85	0.07	1.33	1.12-1.58	0.001
Prior myocardial infarction	1.21	0.79-1.86	0.37	0.83	0.52-1.32	0.44	0.86	0.68-1.09	0.21
No chest discomfort	1.28	0.96-1.71	0.09	1.73	1.27-2.35	0.001	1.29	1.07-1.55	0.01
Renal dysfunction	2.01	1.59-2.52	<0.001	2.10	1.64-2.69	<0.001	2.13	1.84-2.45	<0.001
LBBB	1.54	0.83-2.88	0.17	1.80	0.92-3.56	0.09	1.08	0.68-1.72	0.73
Cardiac arrest	0.43	0.13-1.43	0.17	5.47	2.65-11.28	<0.001	1.99	1.22-3.25	0.01
Cardiogenic shock	1.78	1.20-2.66	0.01	2.03	1.37-3.02	0.001	2.02	1.62-2.53	<0.001
Acute stroke	1.80	1.09-2.98	0.02	1.83	1.11-3.03	0.02	3.01	2.24-4.04	<0.001
Heart rate >90 beats/min	1.52	1.21-1.91	<0.001	1.68	1.32-2.13	<0.001	1.66	1.45-1.91	<0.001
SBP <100 mm Hg	1.48	1.08-2.03	0.02	1.55	1.10-2.18	0.01	1.54	1.25-1.90	<0.001
White blood cell count									
6000/μL-12 000/μL	0.97	0.71-1.33	0.86	1.18	0.86-1.63	0.30	0.94	0.78-1.13	0.50
>12 000/μL	1.96	1.40-2.74	<0.001	2.18	1.55-3.07	<0.001	2.04	1.66-2.49	<0.001
Receiving all treatments[a]	0.50	0.37-0.67	<0.001	0.49	0.35-0.69	<0.001	0.62	0.52-0.74	<0.001

CHD indicates coronary heart disease; CI, confidence interval; LBBB, left bundle branch block; OR, odds ratio; SBP, systolic blood pressure.

[a] Use of treatment determined among ideal patients.

© 2020 Zhong Q et al. *JAMA Network Open*.

eTable 10. Association Between Utilization of All Treatments and 5-day Mortality in Different Regions

Characteristics	5-day Mortality								
	Western			Central			Eastern		
	OR	95% CI	P value	OR	95% CI	P value	OR	95% CI	P value
Age, every 5 years	1.21	1.13-1.29	<0.001	1.23	1.15-1.32	<0.001	1.25	1.20-1.30	<0.001
Female	1.69	1.28-2.23	<0.001	1.57	1.16-2.13	0.004	1.42	1.19-1.70	<0.001
Hypertension	0.89	0.68-1.17	0.41	0.88	0.66-1.17	0.37	0.79	0.66-0.94	0.01
Diabetes	1.44	1.05-1.98	0.03	1.51	1.07-2.14	0.02	1.15	0.94-1.39	0.18
Dyslipidemia	0.87	0.47-1.60	0.65	0.62	0.29-1.31	0.21	0.93	0.65-1.33	0.68
Current smoker	0.88	0.63-1.22	0.45	0.87	0.59-1.28	0.47	0.95	0.77-1.18	0.65
History of CHD	0.96	0.65-1.43	0.86	0.94	0.65-1.37	0.76	1.25	1.01-1.54	0.04
Prior ischemic stroke	1.36	0.93-1.99	0.11	1.29	0.87-1.91	0.20	1.13	0.90-1.41	0.29
Prior myocardial infarction	1.14	0.66-1.95	0.65	0.93	0.53-1.63	0.79	0.76	0.56-1.03	0.07
No chest discomfort	1.10	0.78-1.56	0.58	1.71	1.18-2.46	0.004	1.15	0.91-1.45	0.24
Renal dysfunction	1.68	1.28-2.21	<0.001	2.10	1.56-2.84	<0.001	2.02	1.69-2.42	<0.001
LBBB	0.68	0.25-1.83	0.45	1.97	0.91-4.27	0.09	0.93	0.52-1.67	0.82
Cardiac arrest	0.20	0.03-1.39	0.11	2.87	1.13-7.30	0.03	2.23	1.29-3.85	0.004
Cardiogenic shock	2.20	1.41-3.44	0.001	2.31	1.48-3.59	<0.001	1.99	1.53-2.58	<0.001
Acute stroke	2.36	1.36-4.09	0.002	1.46	0.77-2.77	0.24	2.75	1.93-3.90	<0.001
Heart rate >90 beats/min	1.40	1.06-1.84	0.02	1.62	1.21-2.16	0.001	1.71	1.44-2.04	<0.001
SBP <100 mm Hg	1.49	1.03-2.15	0.04	1.55	1.04-2.30	0.03	2.00	1.58-2.53	<0.001
White blood cell count									
6000/μL-12 000/μL	1.28	0.87-1.90	0.21	1.03	0.71-1.50	0.88	0.86	0.68-1.08	0.20
>12 000/μL	2.21	1.45-3.38	<0.001	1.79	1.19-2.67	0.01	1.89	1.48-2.42	<0.001
Receiving all treatments[a]	0.41	0.28-0.59	<0.001	0.27	0.16-0.45	<0.001	0.57	0.45-0.73	<0.001

CHD indicates coronary heart disease; CI, confidence interval; LBBB, left bundle branch block; OR, odds ratio; SBP, systolic blood pressure.

[a] Use of treatment determined among ideal patients.

© 2020 Zhong Q et al. *JAMA Network Open*.

eTable 11. Ideal Candidates for Treatments among Patients with Acute Myocardial Infarction

Process of Care[a]	2001-2006			P Value[b]	2011-2015			P Value[b]
	Western	Central	Eastern		Western	Central	Eastern	
Reperfusion[c], n (%)	449 (38.3)	471 (35.4)	1686 (39.6)	0.03	1502 (34.1)	1667 (31.9)	3445 (32.3)	0.05
Aspirin, n (%)	1049 (89.4)	1179 (88.7)	3855 (90.4)	0.16	3769 (85.6)	4435 (84.8)	9217 (86.5)	0.01
Clopidogrel, n (%)	1045 (89.1)	1160 (87.3)	3759 (88.2)	0.38	3787 (86)	4449 (85.1)	9254 (86.9)	0.01
β-blocker, n (%)	498 (42.5)	528 (39.7)	1685 (39.5)	0.19	1810 (41.1)	1997 (38.2)	4339 (40.7)	0.003
ACEI/ARB, n (%)	1035 (88.2)	1180 (88.8)	3806 (89.3)	0.58	3690 (83.8)	4337 (83)	8994 (84.4)	0.06
Statin, n (%)	1070 (91.2)	1203 (90.5)	3945 (92.5)	0.04	3878 (88.1)	4537 (86.8)	9494 (89.1)	<.001
Composite, n (%)	5146 (73.1)	5721 (71.8)	18736 (73.3)	0.03	18436 (69.8)	21422 (68.3)	44743 (70)	<.001

ACEI indicates angiotensin-converting enzyme inhibitor; ARB, angiotensin receptor blocker.

[a] Use of treatment was calculated among ideal patients.

[b] Test for difference among the western, central, and eastern regions.

[c] Reperfusion rate was calculated among patients with ST-elevation myocardial infarction

© 2020 Zhong Q et al. *JAMA Network Open*.
